# Improving stability of elastic stable intramedullary nailing in a transverse midshaft femur fracture model: biomechanical analysis of using end caps or a third nail

**DOI:** 10.1186/s13018-015-0239-z

**Published:** 2015-06-25

**Authors:** Marion Rapp, Nina Gros, Gregor Zachert, Maaike Schulze-Hessing, Christina Stratmann, Robert Wendlandt, Martin Michael Kaiser

**Affiliations:** Department of Paediatric Surgery, Hospital of Kassel, Mönchebergstr. 41-43, 34125 Kassel, Germany; Department of Paediatric Surgery, University of Lübeck, Ratzeburger Allee 160, 23538 Lübeck, Germany; Department of Biomechatronics and Academic Orthopaedics, University of Lübeck, Ratzeburger Allee 160, 23538 Lübeck, Germany

**Keywords:** Elastic stable intramedullary nailing, Biomechanical testing, Femur fracture, End caps, Third nail

## Abstract

**Background:**

Elastic stable intramedullary nailing (ESIN) is accepted widely for treatment of diaphyseal femur fractures in children. However, complication rates of 10 to 50 % are described due to shortening or axial deviation, especially in older or heavier children. Biomechanical *in vitro* testing was performed to determine whether two modified osteosyntheses with end caps or a third nail could significantly improve the stability in comparison to classical elastic stable intramedullary nailing in a transverse femur fracture model.

**Methods:**

We performed biomechanical testing in 24 synthetic adolescent femoral bone models (Sawbones®) with a transverse midshaft (diaphyseal) fracture. First, in all models, two nails were inserted in a C-shaped manner (2 × 3.5 mm steel nails, prebent), then eight osteosyntheses were modified by using end caps and another eight by adding a third nail from the antero-lateral (2.5-mm steel, not prebent). Testing was performed in four-point bending, torsion, and shifting under physiological 9° compression.

**Results:**

The third nail from the lateral showed a significant positive influence on the stiffness in all four-point bendings as well as in internal rotation comparing to the classical 2C configuration: mean values were significantly higher anterior-posterior (1.04 vs. 0.52 Nm/mm, *p* < 0.001), posterior-anterior (0.85 vs. 0.43 Nm/mm, *p* < 0.001), lateral-medial (1.26 vs. 0.70 Nm/mm, *p* < 0.001), and medial-lateral (1.16 vs. 0.76 Nm/mm, *p* < 0.001) and during internal rotation (0.16 vs. 0.11 Nm/°, *p* < 0.001). The modification with end caps did not improve the stiffness in any direction.

**Conclusions:**

The configuration with a third nail provided a significantly higher stiffness than the classical 2C configuration as well as the modification with end caps in this biomechanical model. This supports the ongoing transfer of the additional third nail into clinical practice to reduce the axial deviation occurring in clinical practice.

## Introduction

Fractures of the femoral diaphysis are the second most frequent location of fractures affecting the lower extremity in children (20–26/100,000 children per year) [1, 2] and comprise 1 to 2 % of all fractures in children [3, 4]. More than two thirds occur in children older than 6 years of age [2, 5]. Following the guidelines of the German Society of Paediatric Surgery, children beyond the age of 3 years should be treated with elastic stable intramedullary nailing (ESIN osteosynthesis) even in complex fractures or children older than 12 years as long as sufficient stability can be achieved [6]. ESIN osteosynthesis is said to produce a rapid recovery and a faster reintegration of children and adolescents and lack possible negative effects of immobilisation compared to conservative treatment, especially in schoolchildren [7, 8]. Yet, clinical studies focused on complications following ESIN osteosyntheses revealed problem rates between 10 and 50 % [9–12]. Most complications were observed as a result of instability in complex fracture types and older children weighing more than 40 kg [9, 13, 14]. Because of these instabilities, other authors used additional immobilisation (e.g. application of a cast), additional screws, and an additional external fixation or recommended submuscular plating or external fixation [10, 15–17].

In finding ways to modify elastic stable intramedullary nailing to gain more stability, we first developed a validated adolescent femur spiral facture biomechanical *in vitro* setting [18]. With this, we found that prebending the nails more than 30° is an essential part of the method [19] and furthermore that steel nails improve stability in contrast to titanium nails [18], which might be the reason for fewer complications of steel nails in clinical practice [12]. Although we could not show any improvement with end caps in our validated spiral fracture biomechanical *in vitro* setting [20], there seemed to be an improvement in stability with the implementation of a third nail under some circumstances [21]. In contrast, Volpon and co-workers described during combined axial-bending tests the TEN + CAP combination to be 8.75 % stiffer than nails alone as well as during torsion tests a 14 % increased stiffness in (rare) distal femoral fractures [22]. Their data tend to be congruent with preliminary clinical results in few patients [23, 24]. Therefore, the aim of this study was to determine the influence of these two interesting and intensively discussed modifications with end caps and a third nail (end caps = 2CEC; third nail = 3E) to improve the stiffness of the classical C-shaped elastic stable intramedullary nailing osteosynthesis (2C) in displaced transverse femoral fractures in all possible stress planes.

## Materials and methods

**Fig. 1:**
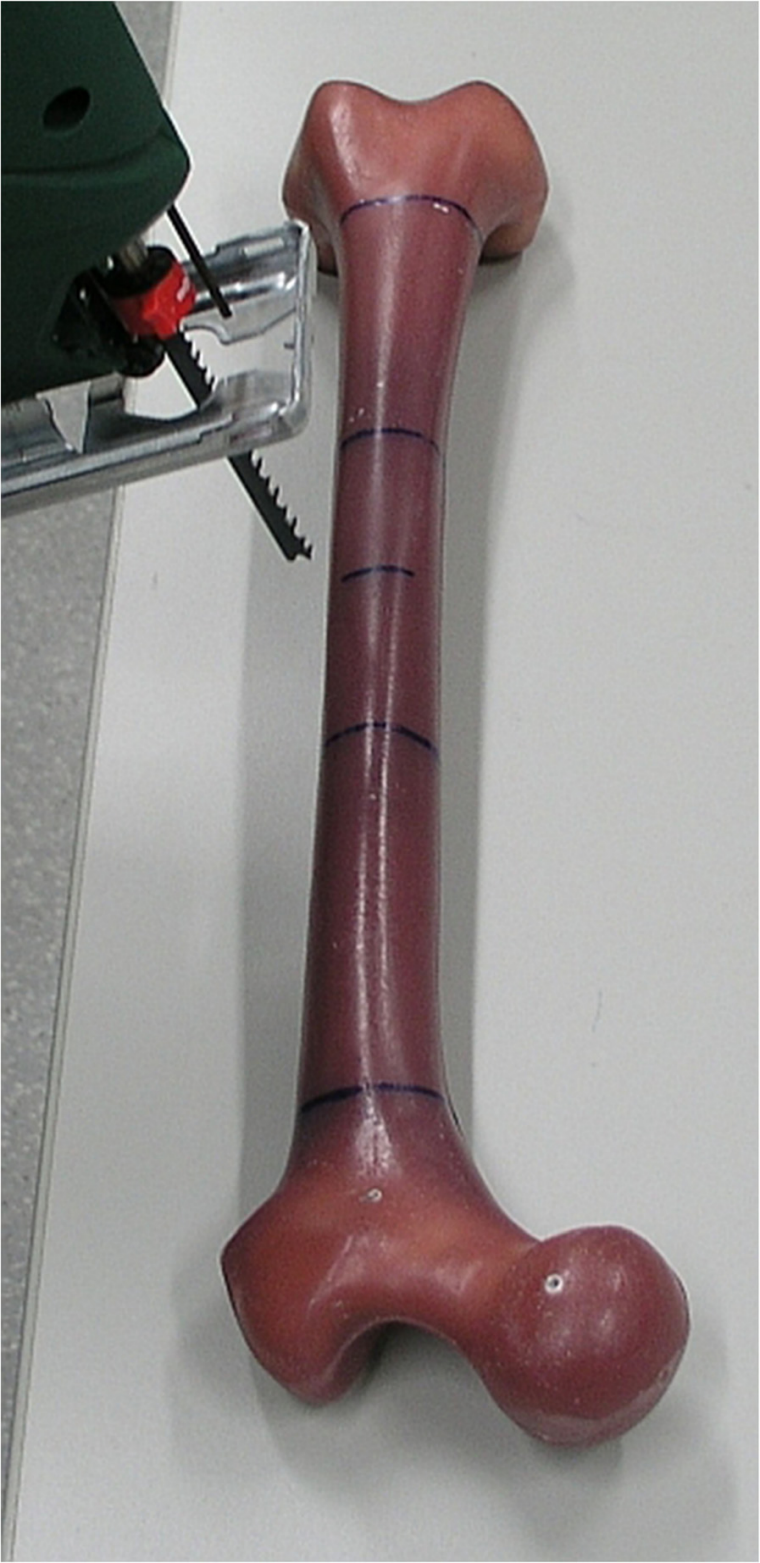
Sawing of a standard midshaft transverse fracture exactly in the middle of the distance between the condyles and trochanter minor (AO paediatric comprehensive classification of long bone fractures: 32D41 [25]; LiLa classification for paediatric long bone fractures: 3.2.s.3.2. [26])

**Fig. 2:**
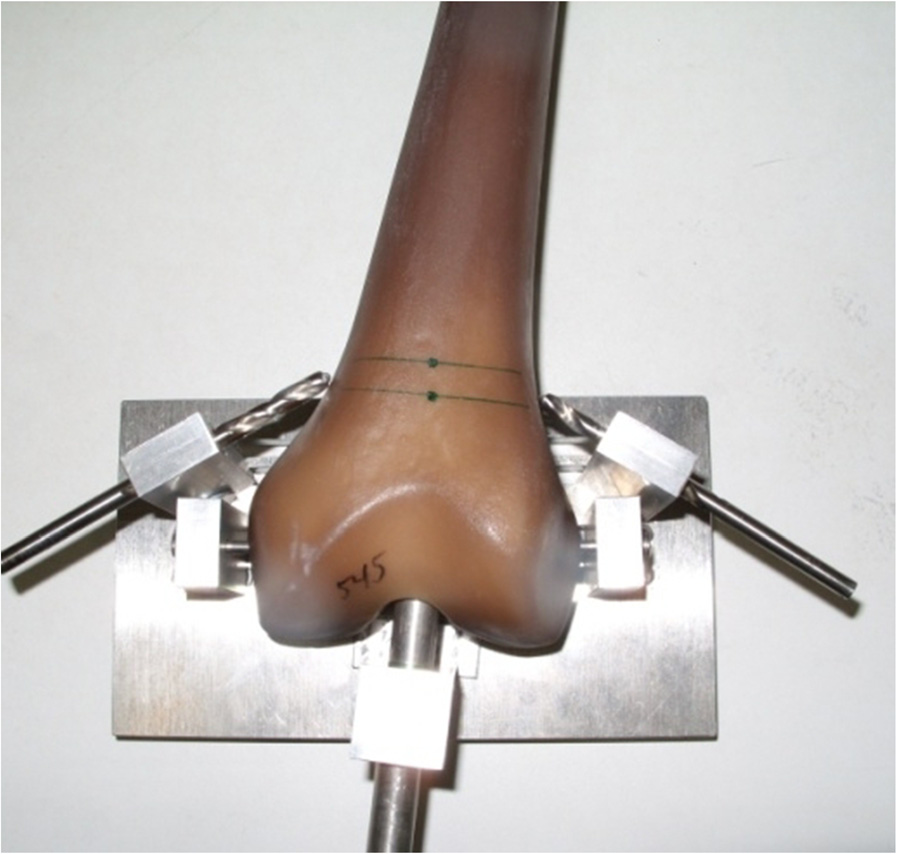
Template for the drilling of the distal femoral entry portals (medial/lateral)

**Fig. 3:**
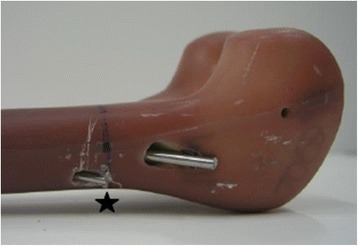
Insertion point for the third nail 2.0 cm cranial anterior of the lateral entry point, the so called “3E” modification

**Fig. 4:**
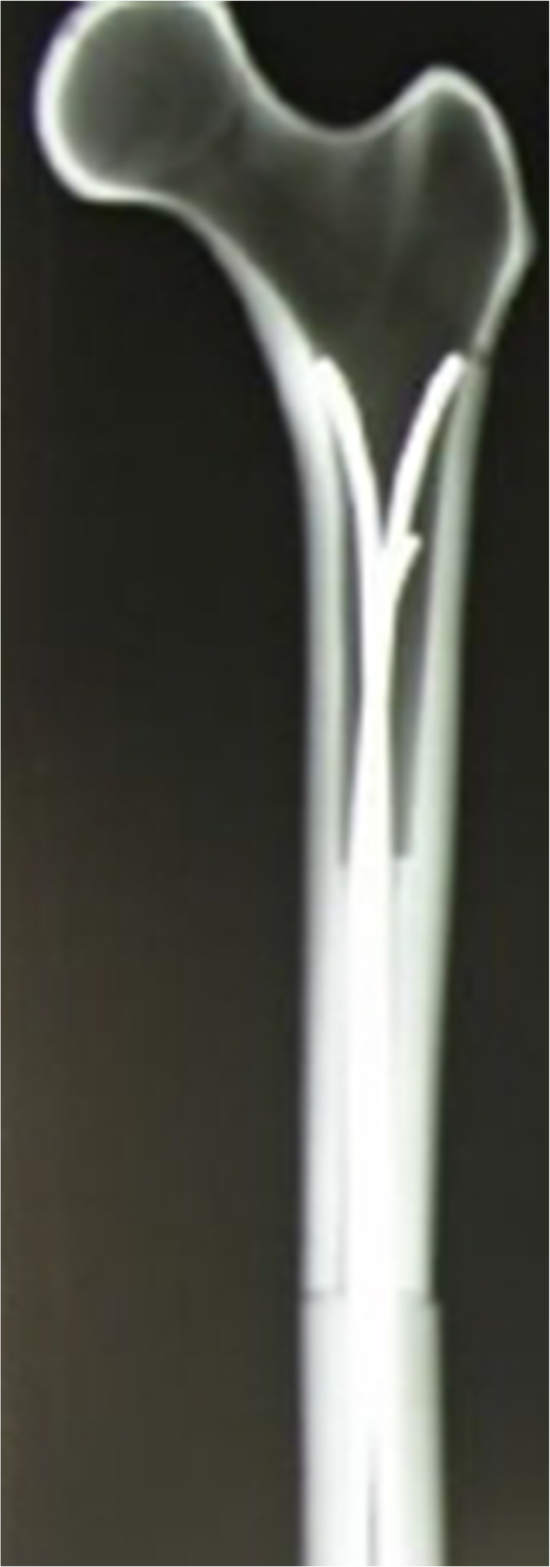
X-ray of the proximal part of the Sawbone with a “3E” modification. Due to the conception of the composite femur, the ends of the nails were just inferior to the greater trochanter

Biomechanical testing was performed using 24 synthetic adolescent-sized composite femoral models (fourth generation, Sawbones®, Malmö, Sweden). The whole setting followed in principle the standardised protocol of previous studies [18]. The femoral model measured 45 cm in length, with a central canal diameter of 10 mm. Each standard midshaft transverse fracture was sawed exactly in the middle of the distance between condyles and trochanter minor (AO paediatric comprehensive classification of long bone fractures: 32D41 [25]; LiLa classification for paediatric long bone fractures: 3.2.s.3.2. [26]) (Fig. 1). The fracture parameters were measured before use in the biomechanical model. The distal femoral entry portals (medial/lateral) were created by a 5-mm drill 2 cm proximal to the virtual physis (Fig. 2). In eight specimens, a further entry point for a third nail was drilled 2 cm cranial anterior of the lateral entry point (Fig. 3). All 24 specimens underwent retrograde elastic stable intramedullary nailing with two 3.5-mm steel nails (Santech Nord, Schneverdingen, Germany), equally prebent to 40°, by the same paediatric surgeon specialised in paediatric traumatology (MMK), with special emphasis on broad contact of the fragments of the transverse fracture. Due to the conception of the composite femur, the ends of the nails were just inferior to the greater trochanter (Fig. 4); fluoroscopic imaging confirmed the correct configuration and position.

**Fig. 5:**
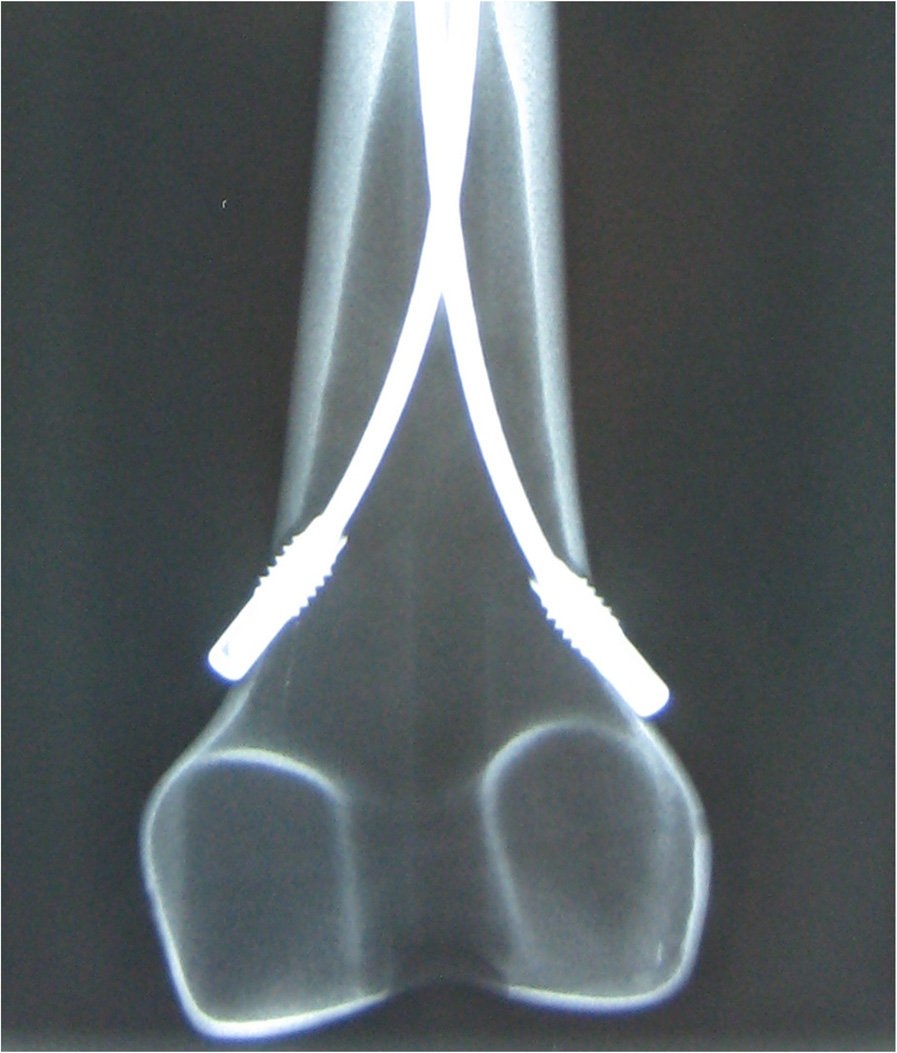
X-ray of the distal part of the Sawbone with a “2CEC” modification
- In the third group (*n* = 8), a third nail (2.5 mm) was inserted over the third entry point from the antero-lateral without prebending, the so-called “3E”-modification (Fig. 3) [21].

The 24 composite models were divided into three configuration groups:

- In the control group (*n* = 8), no further modifications were performed on the two retrograde elastic stable intramedullary nailing configurations (2C).
- In the second group (*n* = 8), two additional cylindric hollow-threaded end caps (green cap for 3.0- to 4.0-mm nail diameters; Synthes Company, Oberdorf, Switzerland) were placed over the external tips of the nails at the entry portals and then screwed into the bone cortex. This modification is further called “2CEC” (Fig. 5).

Testing was done with a Zwick 1465 universal testing machine (UTM; Zwick GmbH & Co. KG, Ulm, Germany). Fixation of the head of the femur and the femoral condyles in the testing machine was achieved with custom-fit polymethylmethacrylate (Technovit 4006, Heraeus Kulzer, Wehrheim, Germany) moulds for both sides. Set-up followed the ASTM F383-73 and F1264-03 description [27, 28].

Initially, the femur was positioned in a 0° position to test for construct stability with a compression load of up to 150 N to the femoral head with a speed of 0.05 mm/s.

**Fig. 6:**
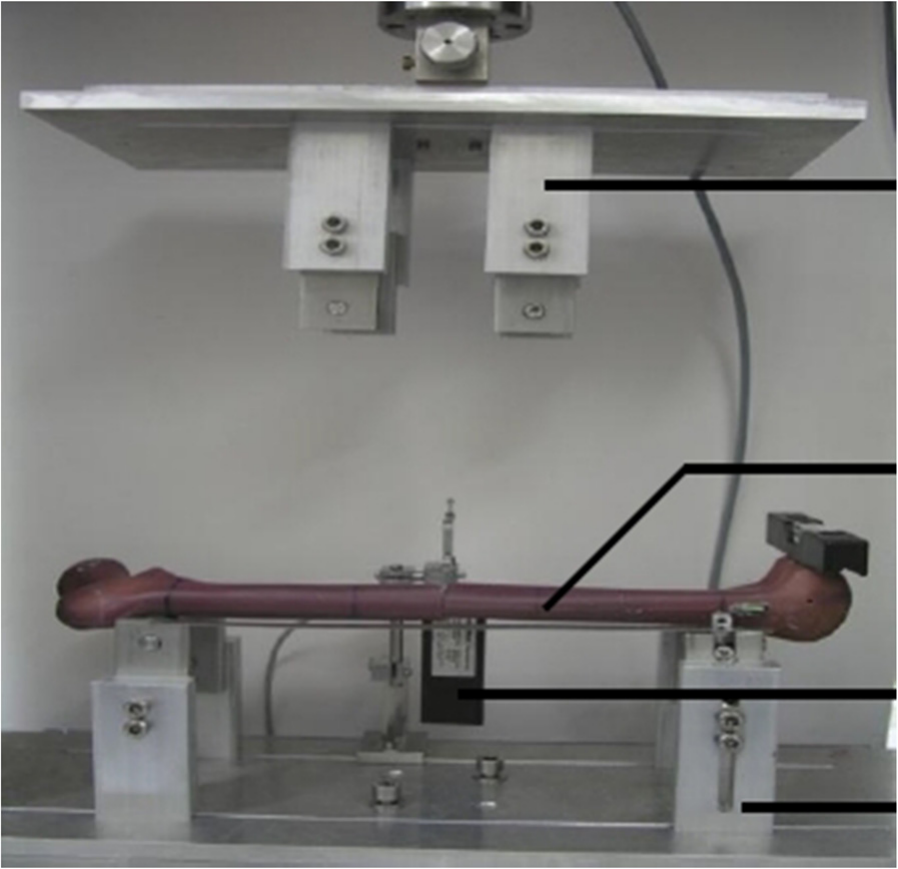
Photograph showing the specimen undergoing the four-point bending test, by using the Zwick 1465 universal testing machine, with the bending measured with a linear encoder. (*arrows* up to down: application of load/Sawbone/sensing device/bearings for the Sawbones)

*Four-point bending* was measured with an incremental linear encoder (MS30-1-LD-2, Megatron, Putzbrunn, Germany) at midpoint of the two lower force bars with a maximum bending moment of 5 Nm (Fig. 6). Speed was set at 0.05 mm/s; maximum bending was 2 mm.

In *torsional testing*, two angular encoders measured the torsion, and the femoral head area was gimbal-mounted. Speed was set at 20°/min; torsion was limited to 10°.

For *shifting testing*, the models were installed in a 9° position with a calibrated wedge. During physiological 9° compression, lateral/medial shifting was measured at the trochanter major, while ventral/dorsal shifting was measured at the crista intertrochanterica. A compressive load of up to 100 N was applied to the femoral head with a speed of 0.05 mm/s. In contrast to our spiral models, the reduction of the fracture gap in the 0 and 9° position was not measured due to direct contact of both fragments [18–20].

The course of the tests was equal to previously published studies: first, each specimen was placed in the machine for the four-point bending tests in a standardised order (anterior-posterior (AP), posterior-anterior (PA), lateral-medial (LM) and finally medial-lateral (ML)), followed by internal (IR) and external (ER) torsional tests and finally shifting tests in the 9° position. The first cycle of the tests was used as preconditioning; data for evaluation were collected from three subsequent cycles. After the last cycle of testing, all specimens were again tested with anterior-posterior bending to check for possible destructive changes that could have influenced the results. Thus, we could exclude destruction of the osteosyntheses and the specimen. Deformation of the UTM was determined up to 50 Nm in pretesting during the four-point bending and did not influence the results.

Data (bending moments in four-point bending, torsional stiffness in IR/ER and shifting in 9° position) were analysed with SPSS 18.0 (SPSS Inc., Chicago, USA). Distributions were first checked for normality (Shapiro-Wilk test); when significant discrepancy from a normal distribution occurred, a Mann-Whitney test was performed. When there was no significant discrepancy from normal distribution, the F-test and analysis of variance (ANOVA) were used. All values are presented as mean values. Significance level was set to *P* < 0.05. Due to multiple testing, the Holm-Bonferroni method was used for *post hoc* comparison.

## Results

All results of the stiffness and the shifting during compression of the three different prebending configurations are shown in Tables 1, 2 and 3. Using end caps did not improve stability in any direction (Table 1). Furthermore, before adjusting the results with the Holm-Bonferroni method, the classical 2C configuration showed greater stiffness in the anterior-posterior and latero-medial as well as less shifting at the crista intertrochanterica in the 0° position. In contrast, the use of a third nail (3E) offered more stiffness in all four-point bendings as well as in the internal and external rotation in comparison to the classical 2C configuration and the modification with end caps (2CEC). Adjusting the results with the Holm-Bonferroni method, the significant differences for the 3C modification mentioned above could all be affirmed, except for the external rotation compared to the classical 2C configuration (Table 2). In comparing the shiftings in the 9° position, the modification with end caps had the highest shifting and was least stable (Table 3).

**Table 1:** Comparison between the stiffness of the osteosyntheses with the 2C classical configuration (2C) and the 2C configuration with end caps (2CEC). Lower changes in shifting tests = higher stiffness

	2C classical (2C)	2C with end caps (2CEC)	*P* value
	(*n* = 8)	(*n* = 7)	
Mean (SD) four-point bending (Nm/mm)			
Anterior-posterior	0.52 (0.49)	0.44 (0.11)	0.04^a^
Posterior-anterior	0.43 (0.11)	0.48 (0.13)	0.89
Lateral-medial	0.70 (0.16)	0.60 (0.19)	0.04^a^
Medial-lateral	0.76 (0.27)	0.73 (0.25)	0.57
Mean (SD) rotation (Nm/°)			
External rotation	0.12 (0.04)	0.10 (0.03)	0.15
Internal rotation	0.11 (0.03)	0.10 (0.03)	0.09
Mean (SD) 9° compression/shifting (mm)			
Shifting 9° trochanter major	0.80 (0.65)	1.77 (1.70)	0.06
Shifting 9° crista intertrochanterica	4.56 (2.67)	7.44 (2.48)	0.01^a^

^a^Adjusting the results with the Holm-Bonferroni method, the significance of the differences could not be confirmed

**Table 2:** Comparison between the stiffness of the osteosyntheses with the 2C classical configuration (2C) and the configuration with a third nail from lateral (3E). Lower changes in shifting tests = higher stiffness

	2C classical (2C)	Third nail from lateral (3E)	*P* value
	(*n* = 8)	(*n* = 8)	
Mean (SD) four-point bending (Nm/mm)			
Anterior-posterior	0.52 (0.49)	1.04 (0.37)	<0.001
Posterior-anterior	0.43 (0.11)	0.85 (0.30)	<0.001
Lateral-medial	0.70 (0.16)	1.26 (0.54)	<0.001
Medial-lateral	0.76 (0.27)	1.16 (0.33)	<0.001
Mean (SD) rotation (Nm/°)			
External rotation	0.12 (0.04)	0.14 (0.02)	<0.01^a^
Internal rotation	0.11 (0.03)	0.16 (0.04)	0.001
Mean (SD) 9° compression/shifting (mm)			
Shifting 9° trochanter major	0.80 (0.65)	1.14 (1.41)	0.98
Shifting 9° crista intertrochanterica	4.56 (2.67)	4.97 (2.89)	0.69

^a^Adjusting the results with the Holm-Bonferroni method, the significance of the differences could not be confirmed

**Table 3:** Comparison between the stiffness of the osteosyntheses with the 2C configuration with end caps (2CEC) and the configuration with a third nail from lateral (3E). Lower changes in shifting tests = higher stiffness

	2C with end caps (2CEC)	Third nail from lateral (3E)	*P* value
	(*n* = 7)	(*n* = 8)	
Mean (SD) four-point bending (Nm/mm)			
Anterior-posterior	0.44 (0.11)	1.04 (0.37)	<0.001
Posterior-anterior	0.48 (0.13)	0.85 (0.30)	<0.001
Lateral-medial	0.60 (0.19)	1.26 (0.54)	<0.001
Medial-lateral	0.73 (0.25)	1.16 (0.33)	<0.001
Mean (SD) rotation (Nm/°)			
External rotation	0.10 (0.03)	0.14 (0.02)	<0.001
Internal rotation	0.10 (0.03)	0.16 (0.04)	<0.001
Mean (SD) 9° compression/shifting (mm)			
Shifting 9° trochanter major	1.77 (1.70)	1.14 (1.41)	0.24
Shifting 9° crista intertrochanterica	7.44 (2.48)	4.97 (2.89)	<0.001

Adjusting the results with the Holm-Bonferroni method, the significance of the differences could all be confirmed

## Discussion

Elastic stable intramedullary nailing (ESIN osteosynthesis) for displaced paediatric femoral fractures in childhood has gained wide acceptance [7, 29], and even older children and sometimes adolescents are treated this way in Europe [6, 10]. On the other hand, publications with special emphasis on problems of this technique revealed complication rates between 10 and 50 % especially—because of residual instability—in complex femoral shaft fractures and in older children or adolescents [9, 10, 12, 30]. To improve elastic stable intramedullary nailing and using stiffness as a marker for stability [31], biomechanical properties of retrograde C-shaped flexible intramedullary nailing are described in the literature [18, 19, 32–36]: Gwyn performed biomechanical testing with different fracture types in synthetic bone models using two titanium elastic nails of 4-mm diameter. The tests were limited to rotational forces, both external and internal, and showed that transverse fractures are as less stable as comminuted fractures with the classical 2 ESIN osteosynthesis [35]. This result was confirmed by Fricka [33]. Most of the other authors focussed on transverse fractures, too, but tested only one or two stress planes without any further explanations [33–36]. In contrast, we are certain that, considering the complex treatment problems, femur fractures require testing in all stress planes as in our previous *in vitro* settings [18–21]. With the aim of achieving further improvement in the ESIN technique, we tested two different modifications (end caps and a third nail), both established clinically in case series [21, 24] but not yet well analysed in a validated biomechanical model of a transverse femoral fracture. Only one study focussed on end caps in a transverse fracture model but in a very distal fracture type, which is a less frequent transverse shaft fracture. In focussing on the impact of end caps and a third nail to improve the stability in ESIN osteosynthesis in a transverse fracture model, this study revealed a benefit towards the configuration with a third nail providing a significantly higher stiffness than the classical 2C configuration. The configuration with a third nail was even stiffer than the modification with end caps which showed no statistical difference to the classical 2C configuration in this transverse biomechanical fracture model. In this biomechanical model, the insertion of the additional third nail revealed no difficulties if the first and second nails of the classical 2C configuration were placed in a technically correct way.

The ongoing transfer of the additional third nail into routine clinical practice in our hospital showed that one has to ensure that the third nail has the least length at the trochanter major and is only used if the first two nails are placed correctly. Then the additional operation time is below 10 min, and the complications due to insufficient stability are reduced [21].

As with every biomechanical study, this one suffers two limitations in comparison to clinical “reality”. The first one includes the use of a synthetic bone model that cannot precisely reproduce all *in vivo* conditions. However, the synthetic bone model has been used successfully in previous biomechanical studies [19, 37] and provides more consistency among specimens than cadaveric bones [38–41]. Also, surrounding tissue like periosteum and muscles were missing which might be an additional stabilising part of the elastic stable fixation method. As in other study groups [34–37], we used a “pure” model without further fixation devices. As reduction of paediatric femoral fractures is almost always performed in a closed manner, we have no precise data about the condition of the periosteum in the case of a fracture which could be used for a more realistic model.

Another limitation was due to the configuration [18, 37]: the ends of the nails could not be placed as proximal as intended in a real procedure. In our opinion, this limitation should be equalised as all three configurations were identically established. On the other hand, during the set-up, the focus was on a consistent surgical technique with an identical and reproducible set-up.

Improper location of the nails or the bends in the nails creates an imbalance in the bending forces, resulting in an angular deformity. This serious technical mistake has been reported in the literature [8]. In our opinion, the proper configuration of the nails was achieved more precisely in the present study than in a real surgical situation.

## Conclusions

The results support the modification of the classical two-C-shaped elastic osteosynthesis in femoral fractures with an additional third nail also in transverse fractures. As feasibility and short implantation time could already be shown in spiral fractures, this treatment improves stability and will help to reduce misalignment or revision surgery.
